# Reconfigurable Josephson Phase Shifter

**DOI:** 10.1021/acs.nanolett.1c01366

**Published:** 2021-06-11

**Authors:** Taras Golod, Razmik A. Hovhannisyan, Olena M. Kapran, Vyacheslav V. Dremov, Vasily S. Stolyarov, Vladimir M. Krasnov

**Affiliations:** † Department of Physics, Stockholm University, AlbaNova University Center, SE-10691 Stockholm, Sweden; ‡ Moscow Institute of Physics and Technology, 141700 Dolgoprudny, Russia

**Keywords:** superconductivity, Josephson junctions, Abrikosov vortices, cryo-electronics

## Abstract

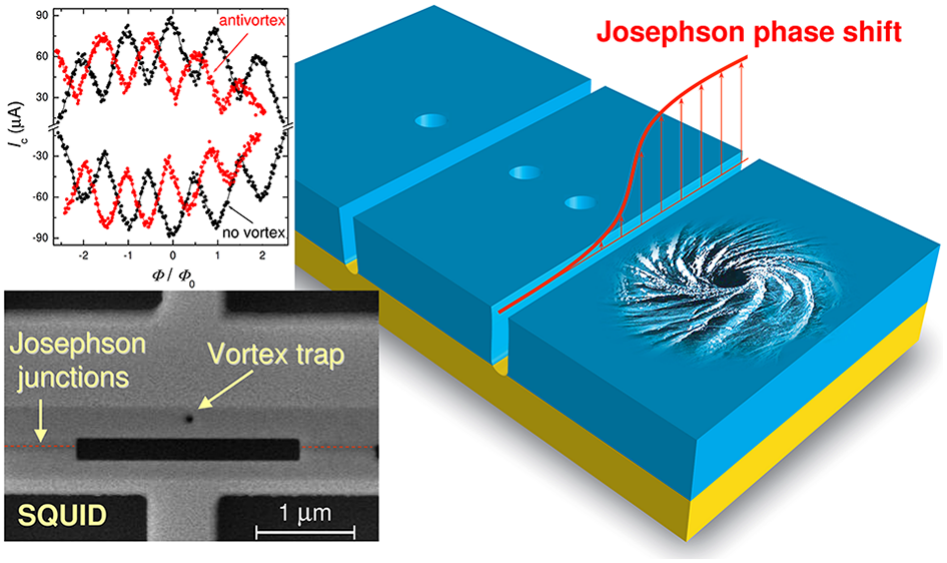

Phase shifter is one of the key elements of quantum electronics. In order to facilitate operation and avoid decoherence, it has to be reconfigurable, persistent, and nondissipative. In this work, we demonstrate prototypes of such devices in which a Josephson phase shift is generated by coreless superconducting vortices. The smallness of the vortex allows a broad-range tunability by nanoscale manipulation of vortices in a micron-size array of vortex traps. We show that a phase shift in a device containing just a few vortex traps can be reconfigured between a large number of quantized states in a broad [−3π, +3π] range.

## Introduction

Electronic devices operate with certain electronic degrees of freedom: conventional electronics with the charge, spintronics with the spin, and quantum electronics with the phase of the electron wave functions. A phase shifter is an important element of quantum devices such as qubits.^1−12^ It can also be used in complementary digital electronics,^13−16^ cryogenic memory,^17−22^ and phase batteries.^23,24^ For most applications, the phase shifter has to be both persistent and tunable. The autonomous (persistent, nondissipative) operation is needed to avoid decoherence in qubits,^4−6^ as well as for realization of complementary digital electronics^13−16^ and nonvolatile memory.^17−20,22^ The tunability is required both for qubit^2,3,7−12^ and for memory operation. Simultaneously, phase shifters should be small, to facilitate downscaling. The combination of persistency, tunability, and compactness is difficult to realize. Depending on the number of achievable states the phase shifter can be switchable between two (0/1) states, reconfigurable between several states, or continuously tunable.

Superconducting electronics typically operates with the Josephson phase difference φ. Usually φ = 0 in the absence of applied current or magnetic field. However, unconventional Josephson junctions (JJs) may provide either a fixed Josephson phase shift (JPS) φ ≠ 0 (φ-junctions) or an in-built spatial phase variation along the JJ φ(*x*) ≠ 0 (0 – φ junctions). The π JPS is most commonly needed, for example, for bringing a qubit to the degeneracy point,^1−4^ for complementary Josephson electronics,^13−16^ and for maximum distinction between 0/1 states in memory cells.^20^ Several ways of creation of JPS are known. A spatial phase variation within JJs can be introduced by inhomogeneities and uneven current distribution.^25−29^ π-junctions with a fixed φ = π phase can be realized using hybrid superconductor/ferromagnet (S/F) structures^30−32^ and unconventional superconductors with a sign-reversal order parameter.^13,33−35^ φ-junctions with an arbitrary JPS can be realized using inhomogeneous SFS JJs,^36,37^ or JJs with a strong spin–orbit coupling.^24^

The majority of phase-shifted JJs are either persistent but not tunable (e.g., SFS and JJs with a sign-reversal order parameter) or tunable but not persistent (e.g., JJs with current injection^26^). The tunability can be achieved in Josephson spin-valve-type devices with several F-layers^38−40^ or in SFS JJs with a strong spin–orbit coupling.^24^ However, it is not clear if SFS JJs are suitable for qubits, because magnetism is considered as one of the main sources of decoherence.^41,42^ Therefore, flux qubits utilize an additional “trap loop” with trapped flux quanta, Φ_0_, nearby the qubit, which offsets the qubit state.^4−6^ The offset flux, Φ, is switchable by magnetic field and adjustable by the loop/qubit geometry. Similar phase shifters have been used for digital RSFQ electronics.^14,15^ Recently it was shown that an Abrikosov vortex can induce a JPS in nearby JJs in the full [−2π, + 2π] range.^23,43,44^ Since vortices can be easily manipulated (displaced, introduced, or removed) by magnetic field, *H*,^23,45−47^ current, *I*,^20,48,49^ and light,^50,51^ they can be used for creation of memory cells^20^ and tunable phase shifters.

Here, we present a very simple realization of a persistent, reconfigurable, and compact Josephson phase shifter. Our approach is based on manipulation of quantized superconducting vortices in an array of few traps. A vortex is the most compact magnetic object in superconductors with the size ∼100 nm. The small vortex size facilitates broad-range tunability of JPS by nanoscale displacement of vortices. In order to avoid dissipation and decoherence caused by quasiparticles and vortex motion, we employ coreless vortices firmly trapped in an array of nanoscale holes. We demonstrate that a great number of multivortex states can be achieved in a device with only few traps. This leads to controllable and almost continuous reconfigurability of a vortex-based phase shifter in a broad [−3π, +3π] range.

## Results and discussion

**Figure 1:**
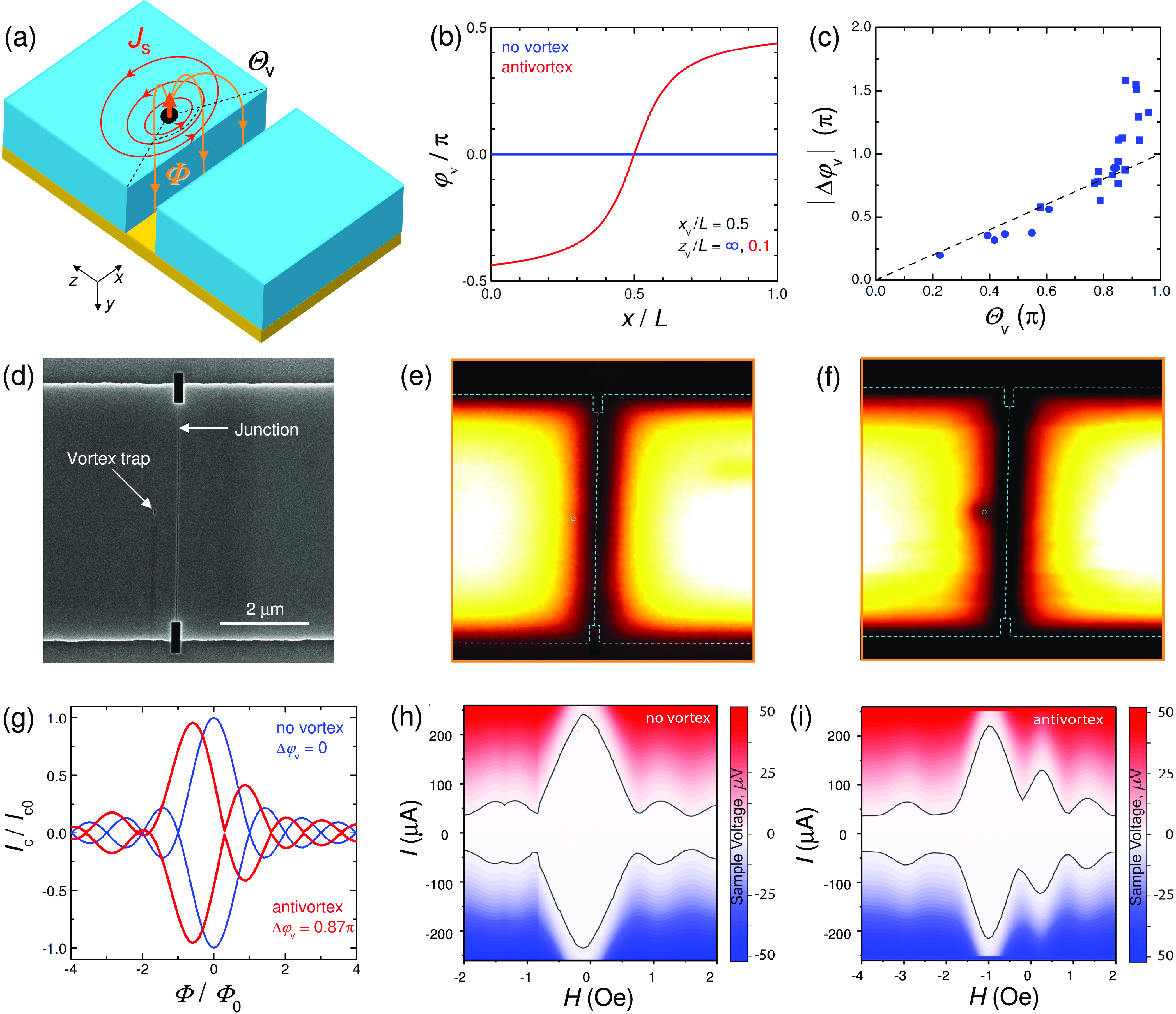
(a) Sketch of a Josephson junction with a trapped vortex in one electrode. Circulating currents and vortex stray fields induce a Josephson phase shift. (b) The red line represents the antivortex-induced JPS, calculated from eq 1 for *z*_v_/*L* = 0.1. The blue line shows the case without the vortex. (c) Measured JPS from a single vortex as a function of vortex polar angle (data from ref (44)). (d) SEM image of a device with one planar junction and a single vortex trap at *z*_v_ ≃ 0.1*L*. (e,f) MFM images (phase maps) of the device without (e) and with (f) a trapped antivortex. (g) Calculated *I*_c_(Φ) modulation patterns for phase shifts from (b) without (blue) and with (red) an antivortex. (h,i) Measured *I*_c_(*H*) modulations for the device in the absence of the vortex (h) and with a trapped antivortex (i). It is seen that the distortion of the *I*_c_(*H*) pattern due to vortex-induced JPS in (i) is consistent with the corresponding simulation in (g).

Figure 1a shows a sketch of a JJ with a trapped antivortex in one of junction electrodes. The vortex has circulating currents (shown by red lines) and stray magnetic fields spreading outside the vortex (shown by orange lines). They create two mechanisms of JPS generation.^44^ The vortex-induced JPS, φ_v_, depends on the distance to the junction, *z*_v_, and the position, *x*_v_, along the junction. As shown in ref (44), it is well described by a steplike function:
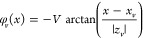
1

Here *V* is the vorticity, +1 for a vortex [magnetic field in the positive *y*-axis direction, as sketched in Figure 1a], −1 for an antivortex. In this case, the total JPS is simply equal to the polar angle, Θ_v_, of the vortex within the junction, Δφ_v_ = φ_v_(*L*) – φ_v_(0) = Θ_v_. The red line in Figure 1b shows corresponding φ_v_(*x*) for an antivortex placed in the middle of the electrode, *x*_v_ = 0.5*L*, at a distance *z*_v_ = 0.1*L* to the JJ. In Figure 1c, we reproduce measured vortex-induced JPS as a function of Θ_*v*_.^44^ It is seen that a single vortex can produce any phase shift in the full range [0, 2π]. In what follows, we utilize this phenomenon for creation of a reconfigurable Josephson phase shifter.

Studied devices contain Nb-based planar JJs.^52^ Details of sample fabrication and experimental procedures can be found in the Supporting Information. First, we demonstrate operation of the simplest device containing one planar JJ with *L* ≃ 4.6 μm and one vortex trap (a hole with a diameter 30–50 nm) at *x*_v_ ≃ *L*/2 and *z*_v_ = 500 nm ≃ 0.1*L*. Figure 1d shows a scanning electron microscope (SEM) image of the device. Figure 1e,f shows magnetic force microscopy (MFM) images of the device at *T* ≃ 4.4 K in the absence of the vortex (e) and with a trapped antivortex (f). The antivortex is clearly visible as a dark spot at the trap. Vortex-induced JPS leads to a distortion of the critical current modulation pattern, *I*_c_(*H*).^23,29,43,44^Figure 1g represents numerically simulated *I*_c_(Φ) patterns without a vortex (blue) and for the JPS from panel b, created by an antivortex at *z*_v_ = 0.1*L* (see the Supporting Information). The *I*_c_(*H*) distortion uniquely depends on the JPS and can be used for estimation of φ_v_.^29,44^

Figure 1h shows the *I*_c_(*H*) modulation for this JJ measured in the vortex-free state, Figure 1e. It follows a regular Fraunhofer modulation. Figure 1i shows the *I*_c_(*H*) pattern after trapping an antivortex, Figure 1f. Apparently, the trapped antivortex distorts *I*_c_(*H*), in good agreement with the simulation in Figure 1g (red line), made for the similar geometry. The JPS can be estimated by numerical fitting of experimental *I*_c_(*H*) patterns, as described in refs (27, 29, and 44).

**Figure 2:**
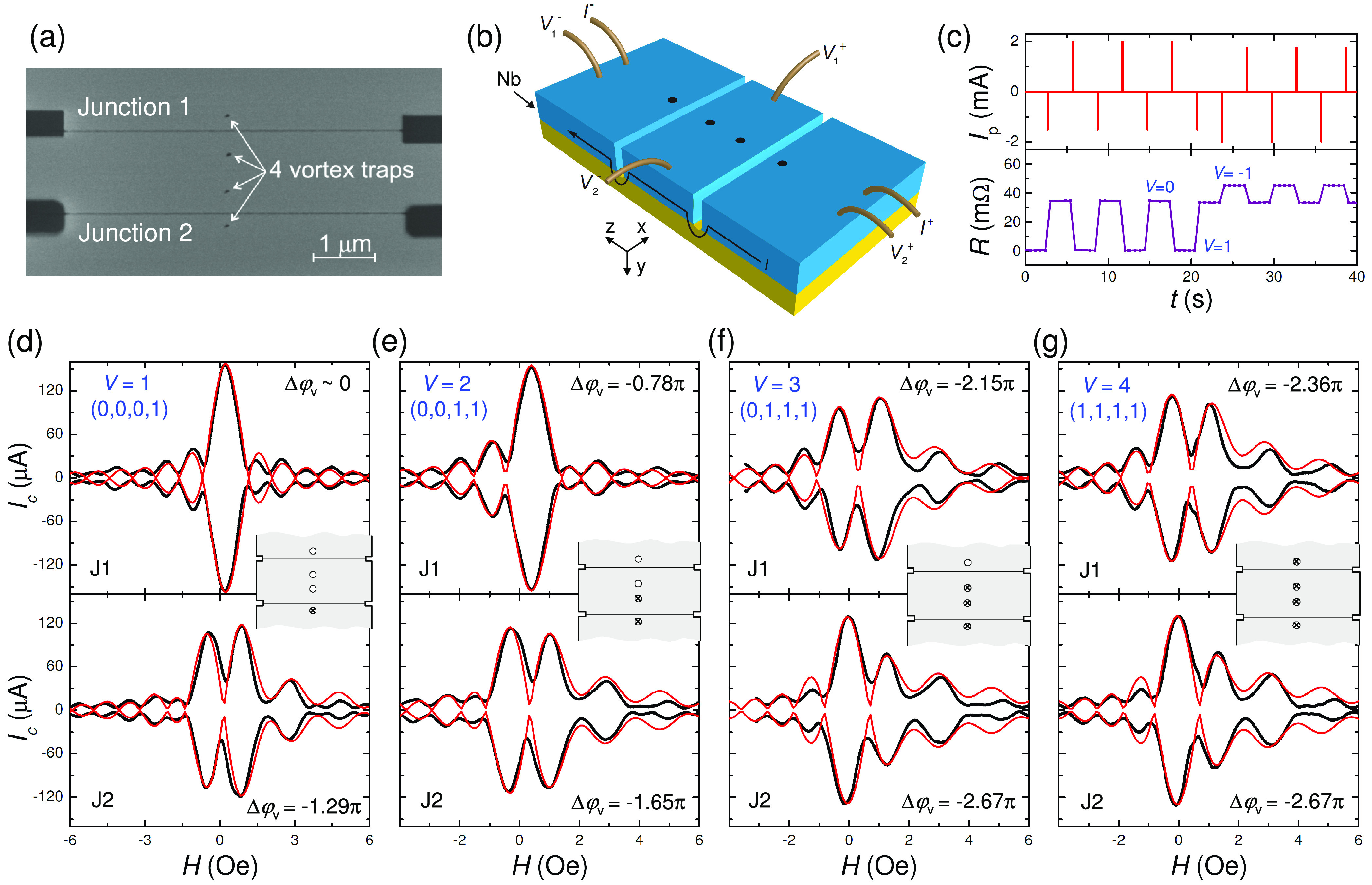
(a,b) SEM image (a) and a sketch (b) of a device with four vortex traps. (c) Demonstration of vortex manipulation by current pulses at *H* = 0. The top panel shows the pulse train with different current amplitudes. The bottom panel shows simultaneously measured junction resistance. Reproducible switching between vortex-free *V* = 0, vortex *V* = 1, and antivortex *V* = −1 states is seen. (d–g) Measured (black) and simulated (red) *I*_c_(*H*) patterns of both junctions on this device for different vortex states with increasing vorticity: (d) (0,0,0,1), (e) (0,0,1,1), (f) (0,1,1,1), and (g) (1,1,1,1). The induced JPS values are indicated in each panel. Insets show corresponding vortex configurations.

Figure 2a shows an SEM image of a more complex prototype of a reconfigurable phase-shifter. It contains two planar JJs and four vortex traps at different locations (exact dimensions are specified in the Supporting Information). Figure 2b represents a sketch of the device. Four electrodes with multiple contacts allow independent measurements of both JJs. The two JJs (J1, J2) are made solely for identification of various vortex states.^44^ Vortices are manipulated (introduced and removed) by short current pulses, *I*_p_.^20^ The top panel in Figure 2c shows a pulse train with different amplitudes at *H* = 0. The bottom panel shows the corresponding time-dependence of the junction resistance. It is seen that we can controllably and reproducibly switch between vortex, *V* = 1, antivortex, *V* = −1, and vortex-free, *V* = 0, states. The same procedure can be done in an applied field *H* ≠ 0, which helps to achieve more complex states. We will identify them as (*V*_1_, *V*_2_, *V*_3_, *V*_4_), where *V*_*i*_ is the vorticity in the trap *i*, counted from top to bottom in Figure 2a, and the total vorticity *V* = |*V*_1_| + |*V*_2_| + |*V*_3_| + |*V*_4_|. Black symbols in Figure 2d–g represent measured *I*_c_(*H*) patterns of both JJs for different single vortex, |*V*_*i*_| ≤ 1, configurations. Red lines demonstrate numerical fits, from which we deduce Δφ_v_.

Figure 2d shows the simplest case, when *I*_c_(*H*) in J1 is practically unaffected, Δφ_v_ ≃ 0, but in J2 there is a significant JPS, Δφ_v_ ≃ −1.29π. This is a manifestation of the (0,0,0,1) state with a vortex in the bottom trap, at a maximum distance from J1. Figure 2e shows the case when there is Δφ_v_ ≃ −0.78π in J1 and −1.65π in J2. It corresponds to the state (0,0,1,1) with vortices in the two lower traps. Figure 2f,g represents correspondingly the (0,1,1,1) state with Δφ_v_ ≃ −2.15π and −2.67π, and the (1,1,1,1) state with Δφ_v_ ≃−2.36π and −2.67π. More details about vortex state identification can be found in ref (44).

**Figure 3:**
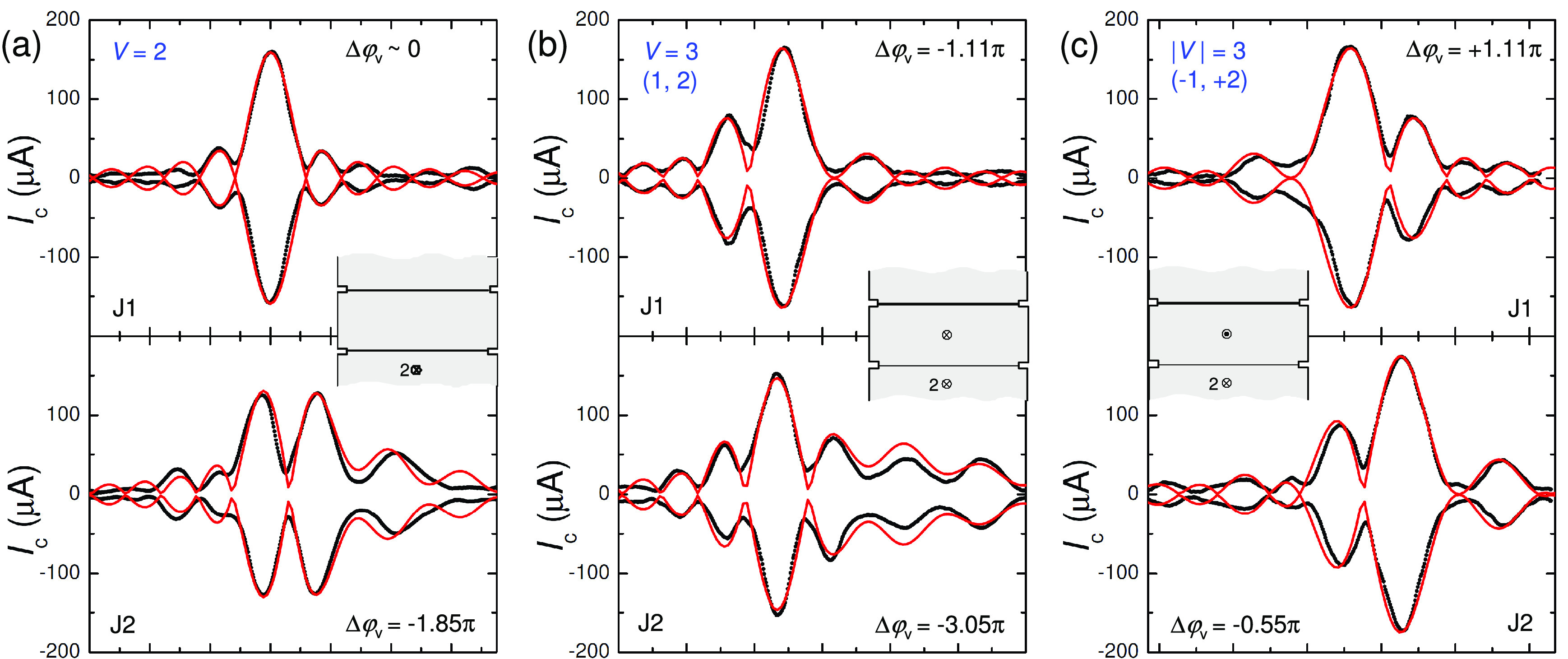
Demonstration of multiquantized vortex states. Measured (black) and simulated (red) *I*_c_(*H*) patterns for another device with two traps. (a) For a doubly quantized vortex in one trap, (b) a doubly quantized vortex in one trap and a single-vortex in another trap, and (c) a doubly quantized vortex and a single antivortex. Insets show corresponding vortex configurations.

At higher magnetic fields more vortices and multiply quantized vortices can be trapped.^53−55^Figure 3 demonstrates states involving a doubly quantized 2Φ_0_ vortex for another device with two vortex traps. Figure 3a shows the state with Δφ_v_ ≃ 0 in J1 and −1.85π in J2. It corresponds to a state with 2Φ_0_-vortex in the bottom trap. Figure 3b corresponds to the case when an additional single Φ_0_ vortex was added in the top hole, resulting in Δφ_v_ ≃ −1.11π in J1 and −3.05π in J2. Finally, Figure 3c represents the state with a double-vortex in the bottom trap and a single antivortex in the top trap. From the presented data, it follows that a large variety of JPS can be induced by changing vortex configurations in a device with only few traps spread over a small distance ∼1 μm. The tunability and the compactness of such devices is directly related to the smallness of the vortex, which allows a significant variation of Δφ_v_ by a nanoscale vortex displacement.

**Figure 4:**
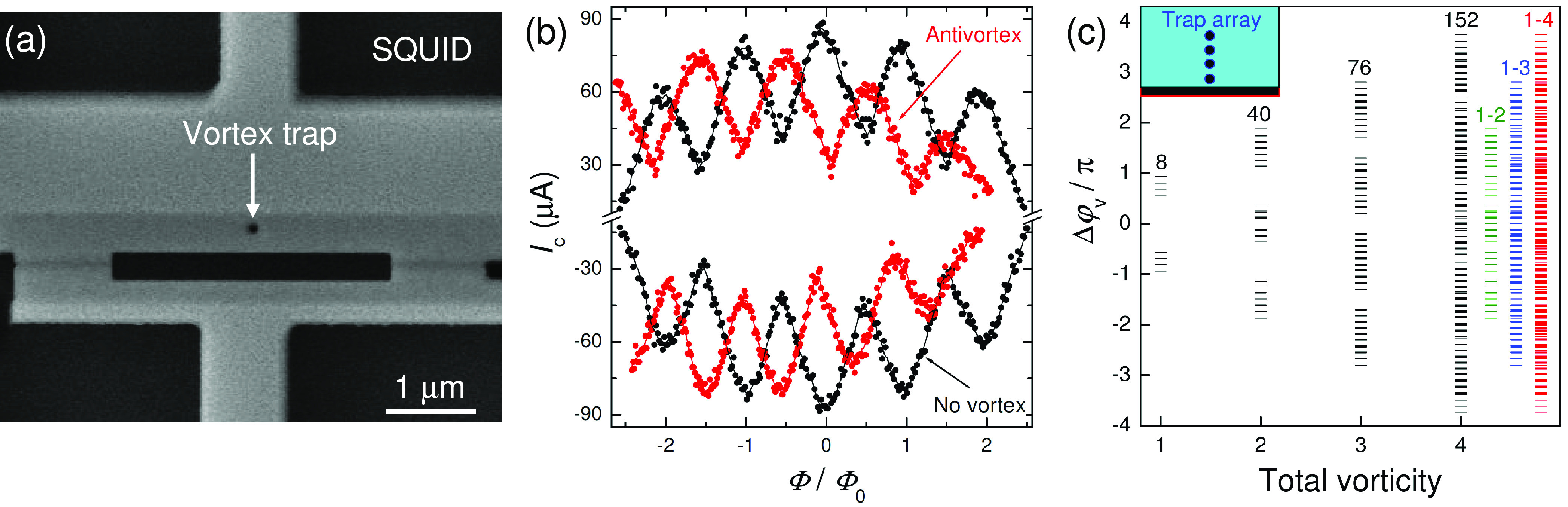
Vortex-induced phase shift in a SQUID. (a) SEM image of a dc-SQUID with a vortex trap. (b) *I*_c_(Φ) modulation of the SQUID without vortex (black) and with a trapped antivortex (red). The half-period shift of patterns indicates that the vortex-induced phase shift is close to π. (c) Theoretical analysis of achievable phase shifts as a function of the total vorticity in a device with four traps, depicted in the inset. Red lines represent the total 276 states with the vorticity 1 ≤ *V* ≤ 4 and indicate an almost continuous broad-range tunability of the phase shifter.

A vortex can induce a phase shift not only in a JJ but also in a device, such as SQUID. Figure 4a represents an SEM image of a dc-SQUID with two JJs and one vortex trap in the top part of the SQUID loop. Figure 4b shows *I*_c_(*H*) modulations of the SQUID without (black) and with (red) an antivortex in the trap. It is seen that the *I*_c_(*H*) modulation patterns are shifted by approximately half a period, indicating that the phase shift is close to π.

Thus, we have demonstrated that vortices can induce JPS in nearby junctions and devices. As shown in Figure 1c, the magnitude of the phase shift is determined by Θ_v_, which depends on the device geometry. As seen from Figure 2, a broad-range reconfigurability can be achieved in a device with just a few vortex traps. In Figure 4c, we present a theoretical analysis of achievable states for a device with four vortex traps, sketched in the inset. The traps are located at *x*_v_/*L*_*x*_ = 0.5 and *z*_vi_/*L*_*x*_ = 0.05, 0.16, 0.27, 0.4. The corresponding polar angles are Θ_vi_ = 0.937π, 0.803π, 0.685π, and 0.570π. We include multiple quantized vortices in the analysis so that each trap could be either empty or contain vortices and antivortices with |*V*_i_| ≤ *V*. For *V* = 1 there are eight states (four vortex and four antivortex) with phase shifts given by ± Θ_vi_. The combinatoric number of states increases rapidly with the total vorticity and reaches 152 for *V* = 4, while the range of the phase shift increases linearly with *V*. Green, blue, and red lines in Figure 4c represent the states with total vorticities *V* ≤ 2, 3, and 4, respectively. In particular, red lines represent 276 states with *V* ≤ 4, which is the sum of the states with *V* = 1, 2, 3, and 4. It is seen that the states are very densely distributed in the range [−3π, + 3π]. Therefore, reconfigurability of such a phase shifter is similar to continuous tunability. However, all of these states are distinct, quantized, and persistent.

A remarkable feature of vortex-based phase-shifters is a combination of the broad JPS range with compactness. From Figure 4c, it follows that a tunability in the [−3π, +3π] range can be achieved via vortex displacements by *z*_v_ < 0.4*L*_*x*_. For *L*_*x*_ = 2 μm, this is only 800 nm. The compactness is facilitated by the small vortex size, ∼100 nm, which enables significant JPS variation by a nanoscale vortex manipulation. This is the main advantage with respect to macroscopic trap loops, employed for offsetting qubits so far.^4−6^ Such loops allow only on–off switching of a certain phase shift, while vortex-based phase-shifters, presented here, can be almost continuously tuned in a broad range without increasing device sizes.

Finally we want to note that Abrikosov vortices are unwanted in quantum devices because they can cause decoherence as a result of extra dissipation in the normal core or due to vortex motion. Here, we employ coreless vortices trapped in holes several times larger than the coherence length of Nb. This leads to a very effective pinning,^45^ immobilizing vortices, and removes vortex cores and associated quasiparticles. Therefore, such vortices should not affect coherence times in quantum devices.

To conclude, we have shown that vortices can be used for creation of reconfigurable Josephson phase shifters. We demonstrated prototypes of such devices in which the phase shift is controlled by geometry (*L*_*x*_), position of vortex traps (*x*_vi_, *z*_vi_, Θ_vi_) and vorticity (*V*_i_). The main advantages of such devices are (i) compactness and (ii) broad range of tunability, facilitated by the small vortex size (vortices represent the most compact magnetic object in a superconductor); (iii) persistent and nonvolatile operation caused by the quantized nature of vortices; (iv) low dissipation and decoherence due to usage of firmly trapped coreless vortices; and (v) easiness of vortex manipulation, which facilitates various ways of operation. We argue that this provides a unique opportunity for creation of compact, tunable, and persistent phase-shifters and phase batteries both for digital and quantum electronics.

## Supporting Information Available

It contains additional clarifications about sample fabrication and geometry, experimental, MFM imaging, and numerical simulations (PDF)
