# Supplementary material for: Reconfigurable Josephson Phase Shifter
Source: Nano Lett. 2021 Jun 11;21(12):5240–6. doi: 10.1021/acs.nanolett.1c01366 (PMC8289326; doi:10.1021/acs.nanolett.1c01366)
Supplement: Supplementary file 1 — nl1c01366_si_001.pdf [file nl1c01366_si_001.pdf]

Supporting information to the manuscript: Reconfigurable Josephson phase shifter

Taras Golod[1], Razmik A. Hovhannisyan[1,2], Olena M. Kapran[1], Vyacheslav V. Dremov[2], Vasily S. Stolyarov[2], and Vladimir M. Krasnov[1,2,*]
[1] *Department of Physics, Stockholm University, AlbaNova University Center, SE-10691 Stockholm, Sweden and*
[2] *Moscow Institute of Physics and Technology (State University), 9 Institutskiy per., 141700 Dolgoprudny, Russia*

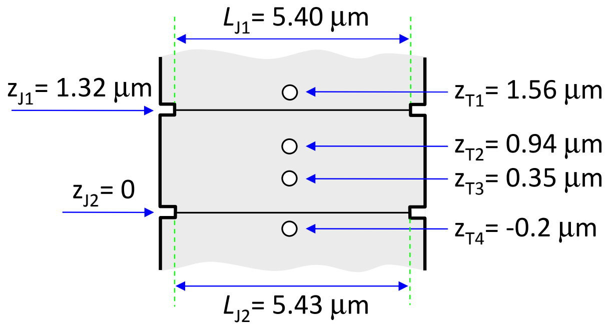

Supplementary Figure 1. Sketch of the geometry of the phase shifter device from Fig. 2 with coordinates of the traps z_{Ti} and junctions z_{Ji} counted from J2.

Samples

Studied devices contain planar JJ's. They are made from bi-layer films with a 70 nm top Nb layer and thin non-superconducting metallic bottom layer. Devices in Figs. 1 (d), 2 and 3 are made using a paramagnetic CuNi alloy, the SQUID from Fig. 4 (a) - using pure Cu underlayer. We also tested other metals and just a single layer Nb film. All of them work in a similar manner and results do not depend on a specific material. Variable-thickness-bridge type JJ's are made by cutting a narrow (∼ 20 nm) grove in the top Nb layer by focused ion beam (FIB) etching, as sketched in Fig. 2 (b). Details of junction fabrication can be found elsewhere [18,23,27]. Planar junction properties were described in Ref. [52]. SQUID device, Fig. 4, was made by FIB milling of a rectangular loop in the middle of a junction.

Supplementary Figure 1 shows a sketch of the device from Fig. 2 with corresponding junction lengths and distances to the traps counted from the bottom junction-2.

Experimental

Measurements are performed in a cryogen-free cryostat using a four-probe configuration. Magnetic field is applied perpendicular to the films. Vortex states are prepared in the following way. We start from the Meissner state by zero-field cooling of a device without bias current. Vortices are introduced either by applying current pulses, magnetic fields, or both, as described in Ref. [18]. Depending on the amplitude and the sign of current pulses, we can introduce either vortices, or antivortices as shown in Fig. 2 (c).

MFM imaging

Low-temperature MFM imaging is carried out on AttoCube scanning probe system (AttoDry 1000/SU) with a standard Co/Cr-coated cantilever (MESP, Bruker, 2.8 N/m spring constant). MFM images, shown in Figs. 1 (e) and (f) are made in a tapping mode at a fixed resonance frequency, ≃ 87 kHz. The color scale represents the phase of tip oscillations: the black color corresponds to zero phase, brighter areas to a positive phase with the brightest level ≃ +10°. The positive phase shift indicates a repulsive force on the tip, which is caused by Meissner screening of the tip field by the superconductor. To trap a vortex, the tip was approached close enough to the hole so that inhomogeneous magnetic field of the tip locally introduced a vortex. The sign of the vortex depends on the direction of tip magnetization. Because the vortex is introduced by the tip field, the tip-vortex interaction is attractive, resulting in the dark contrast of the trapped vortex in a subsequent MFM phase map, shown in Fig. 1 (f). $I_c(H)$ measurements, presented in Figs. 1 (h) and (i) are performed in the same MFM system with a retracted tip, in order not to induce extra distortion from the tip itself [29].

Numerical simulations

We use numerical fitting for extraction of JPS. Simulations presented by red lines in Figs. 1 (g), 2 (d-g) and 3 are done taking $\varphi_v(x)$ from Eq. (1) with actual trap geometries (x_{vi}/L_x, z_{vi}/L_x, Θ_{vi}) and using V_i as a fitting parameter. The critical current is calculated by maximization of integrated Josephson current, $I = (I_{c0}/L)\int_0^L \sin[\varphi(H) + \varphi_v]dx$, where $\varphi(H)$ represents the linear field-dependent phase gradient in the absence of vortices. Details of the formalism can be found in Ref. [29]. In all demonstrated cases such fitting allows unambiguous estimation of JPS, $\Delta\varphi_v = -\sum_i V_i\Theta_{vi}$.

* Corresponding author. E-mail: Vladimir.Krasnov@fysik.su.se
